# Use of GC-IMS for detection of volatile organic compounds to identify mixed bacterial culture medium

**DOI:** 10.1186/s13568-022-01367-0

**Published:** 2022-03-04

**Authors:** Yanyi Lu, Lin Zeng, Min Li, Bowen Yan, Dandan Gao, Bangfu Zhou, Weiping Lu, Qinghua He

**Affiliations:** 1 Department of Military Medical Equipment, State Key Laboratory of Trauma, Burns and Combined Injury, Research Institute of Surgery, Army Medical University, 400042 Chongqing, China; 2 Clinical Laboratory, Daping Hospital, Army Medical University, 400042 Chongqing, China

**Keywords:** GC-IMS, Headspace gas, Identification of bacteria, mVOCs

## Abstract

In order to explore the possibility to identify common wound infection bacteria in mixed culture with gas chromatograph-ion migration spectroscopy (GC-IMS), the headspace gas of single and mixed cultures of *Escherichia coli*, *Staphylococcus aureus* and *Pseudomonas aeruginosa* were detected and analyzed by GC-IMS system. The bacteria were cultured in thioglycolate medium tubes then transferred to the sampling bottles (indirect method), or directly cultured in the sampling bottles (direct method) to allow accumulation of volatile compounds and facilitate automation. The specific microorganism volatile organic compounds (mVOCs) of the three bacteria were obtained. Some of them have been known to certain substance, for example, ethanol, isoamyl acetate, Phenylacetaldehyde, 2-heptanone etc., while others have not. Principal component analysis (PCA) showed that a higher separability can be achieved with direct method than indirect method. This work indicated that it is possible to identify compound bacteria by detecting specific mVOCs with GC-IMS, and the specific mVOCs should be medium-dependent.

## Introduction

Wound infection is defined as the presence of replicating microorganisms within a wound leads to tissue injury, pain, or even septic death in severe cases (Haesler and Ousey 2018; White 2009). Some chronic wound infections can also bring more hurt and outcomes to patients (Cutting et al. 2004). With the increase of antibiotic resistance, the key to treatment is timely use of the right antibiotics for the specific infecting microorganism. Therefore, early and rapid identification of the infecting microorganism is crucial for the opportune implementation of appropriate treatment (Cadogan et al. 2011).

Conventional procedures for identifying pathogenic microorganisms rely on reactions in tubed medium and observation of physical characteristics, such as colonial morphology and odor, coupled with results of Gram staining, agglutination tests, and anti-microbial susceptibility profiles, etc. (Carter and Cole 2012; Jorgensen et al. 2015). New technologies relying on microorganisms’ biochemical characteristics, fatty acid patterns, and/or other metabolic properties further accelerated identification of pathogenic microorganisms (Jorgensen et al. 2015). Since 2010, matrix assisted laser desorption/ionization-time of flight mass spectrometry (MALDI-TOF MS) has become a common method for microbial identification in many advanced clinical laboratories for its advantages of rapidness, usually takes only a few minutes to identify species of different microorganisms (Carbonnelle et al. 2011; Li et al. 2019; Váradi et al. 2017). In addition, nucleic acid-based approaches, typically used for bacterial and fungal identification when biochemical and/or proteomic strategies failed. Current clinical procedures to identify microorganism are mainly based on available instruments, type of infection, and type of specimen. These procedures have been greatly simplified and automated in recent decades, yet the time required for microorganism amplification and biochemical reactions remains significant. Although some of these tests are performed within minutes, complete identification mostly takes over 18 h as culture is needed for a large number of cases (Váradi et al. 2017), during which, the infection may greatly aggravate and become much more difficult to control.

Over the years, several technologies have been tried for rapid identification of microorganisms by detecting their volatile organic compounds (VOCs), including electronic nose (Seesaard et al. 2020; Yusuf et al. 2015), high field asymmetric ion mobility spectrum (FAIMS) (Sun et al. 2019), multi-capillary column-ion mobility spectrometry (MCC-IMS) (Jünger et al. 2012; Kunze et al. 2013; Perl et al. 2011), gas chromatograph-ion migration spectroscopy (GC-IMS) (Daulton et al. 2019; Drees et al. 2019; Langejuergen et al. 2015; Sethi et al. 2013), etc. Among them, MCC-IMS and GC-IMS are especially useful for their extremely high sensitivity to identify and quantify specific VOCs. Compared to MCC, GC uses one single long chromatographic column instead of multiple capillary columns for pre-separation, which is slower but has higher resolution. The microorganism characteristic volatile organic compounds (mVOCs) spectrum may be used for their identification of the microorganisms (Perl et al. 2011). For example, MCC-IMS has been used to test the head-space air of various bacterial cultures, and successfully identified the bacteria within 24 h (Jünger et al. 2012). When MCC-IMS is used to analyze the head-space air of pathogen cultures, it would be most likely to identify the pathogens during their later stages of growth (Kunze et al. 2013). Gas chromatograph coupled to an ion mobility spectrometer (GC-IMS) has also been successfully used for identification of three bacteria cultured in blood cultures (BC) based on their mVOCs spectrum (Drees et al. 2019). The identification was done before colorimetric indicators were available, suggesting the potential application of mVOCs for rapid identification of blood stream infection (BSI). The high sensitivity of GC-IMS even made it possible to use wound swabs instead of wound culture for analysis of mVOC, which greatly accelerates the identification process (Daulton et al. 2019). Their method could detect the presence of infection; however, they did not attempt to separate the different wound infection types.

The above studies suggested that with the high sensitivity of GC-IMS, it’s possible to obtain specific mVOCs patterns of some common wound infection bacteria. However, clinical infections are often complex, involving multiple bacteria, and the mVOCs patterns of the bacteria may be affected by each other, adding an extra level of complexity to identification of infecting bacteria with this method.

In this study, the headspace of mixed bacterial cultures was analyzed with GC-IMS system. For sampling of the headspace, we used both the conventional way-fill some of the culture into the sampling tube, and a more direct way-culture the bacteria in the sampling tube. As far as we know, this is the first study on the possibility to identify common wound-infecting bacteria in mixed culture using GC-IMS.

## Materials and methods

### GC-IMS system

**Fig. 1:**
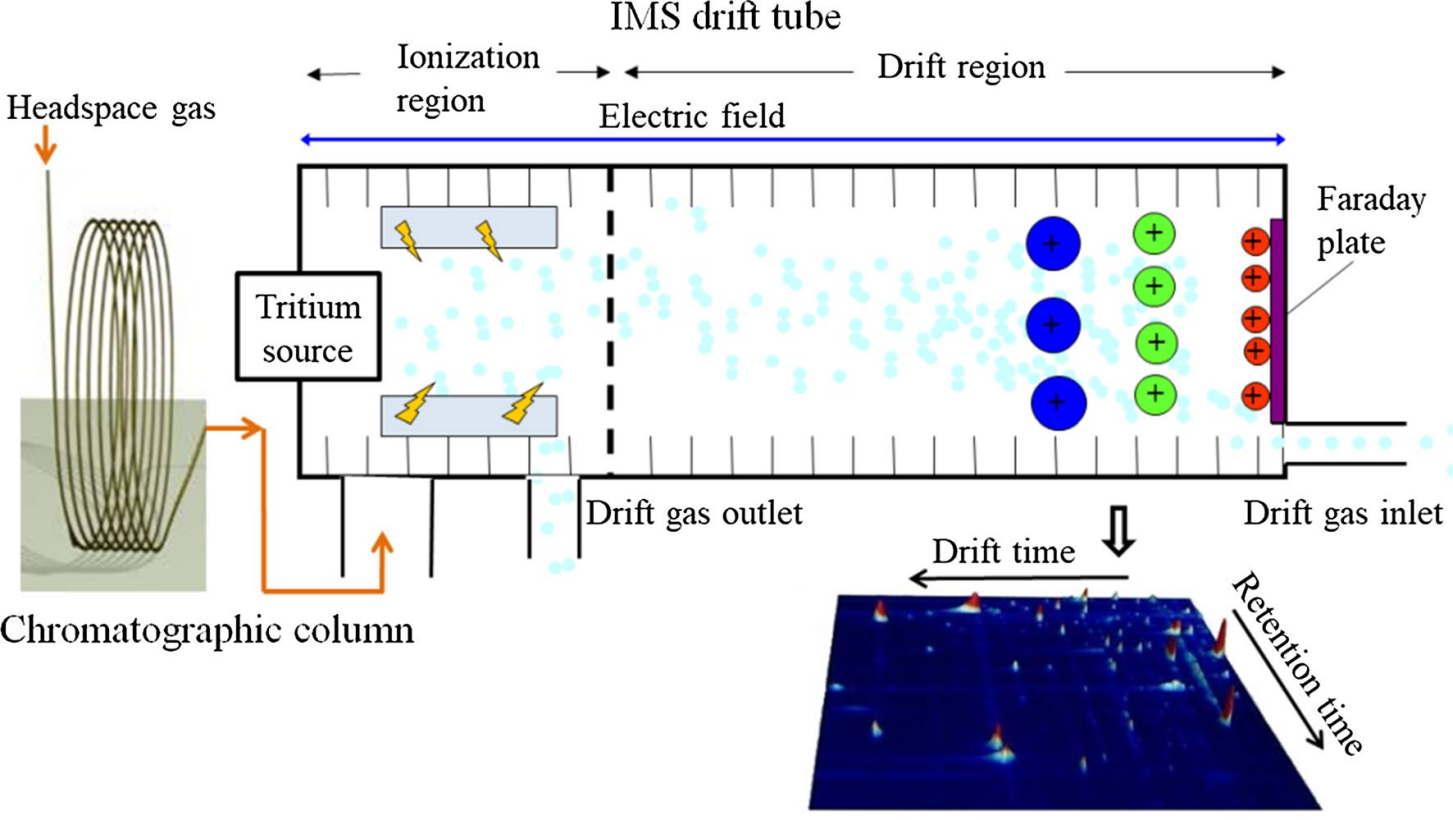
The schematic diagram of GC-IMS

GC-IMS combines the high resolution of GC for pre-separation and high sensitivity of IMS. Molecules are first separated by the GC component due to differences in their interaction with the stationary phase coating on the wall of the chromatographic column. The separated molecules are then ionized (in this case by a tritium source) and are moved along the drift tube by an electric field and the drift gas is blown into the drift tube from the opposite direction, molecules with different mass and charge will take different time to travel to the faraday plate. The retention time in GC part and the drift time in the IMS part, together, describe each unique molecule (Daulton et al. 2019). The schematic diagram of GC-IMS is shown in Fig. 1.

The GC-IMS system used in this paper included a core component (G.A.S, Dortmund, Germany) equipped with a wide bore GC column (mxt-5 15 m × 0.53 mm × 1 μm, RESTEK, USA), an automatic sampler (G.A.S, Dortmund, Germany) that integrated incubating, shaking, and heating functions for easier mVOCs sampling, and a nitrogen generator (G.A.S, Dortmund, Germany) to provide carrier gas. Experimental parameters used are shown in Table 1.

**Table 1:** Experimental parameters

Parameters	Values
Incubation Temperature	60.0 °C
Incubation Time	10.0 min
Sampling volume	1 ml
Detecting time	25 min
Temperature of drift tube: T1	45 °C
Temperature of chromatographic column: T2	40 °C
Temperature of injection port: T3	80 °C
Temperature of joints: T4, T5	Depends on the temperature of the front and rear parts
Flow of drift tube: EPC1	150 ml/min (constant)
Flow of chromatographic column: EPC2	Increase gradually, as shown in Fig. 2

**Fig. 2:**
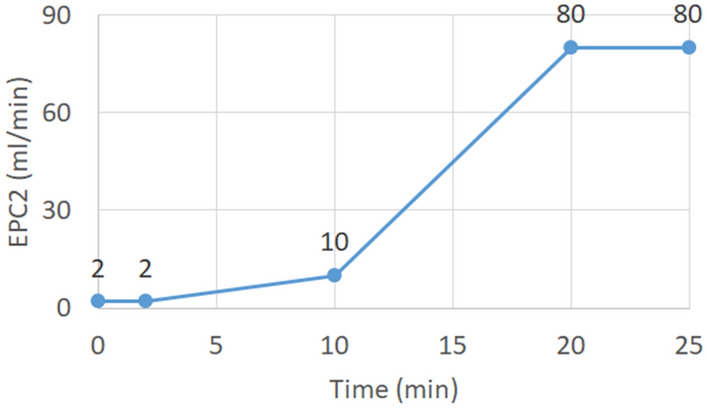
Values of EPC2

The sampling bottles (20 ml) containing the test culture medium were put into the incubator and shaken at 60 °C for 10 min, and then the injection needle takes 1 ml headspace gas from the sampling bottle and injects the sample into the device.

### Culture of bacteria

The three most common bacteria in clinical practice, *Escherichia coli* (ATCC25922, EC), *Staphylococcus aureus* (ATCC25923, SA) and *Pseudomonas aeruginosa* (ATCC27853, PA) were selected as test subjects in this study, which were provided by the clinical laboratory of Daping Hospital, Army Medical University, China. The original bacterial culture solution was properly diluted, applied to the blood agar plate, and cultured for 12-18 h at 37 °C, and then one bacterial colony of each bacterium was picked and cultured in thioglycolate (TH) medium for 12-15 h at 37 °C without agitation before sampling. All 7 combinations of the three bacteria were prepared (EC, SA, PA, EC + SA, EC + PA, SA + PA, EC + SA + PA). TH medium, which was produced by Chongqing Pangtong Medical Instrument Co., Ltd, contains L-cystine, sodium chloride, glucose, yeast extract, casein, trypsin digest and sodium mercaptoglycolate.

**Fig. 3:**
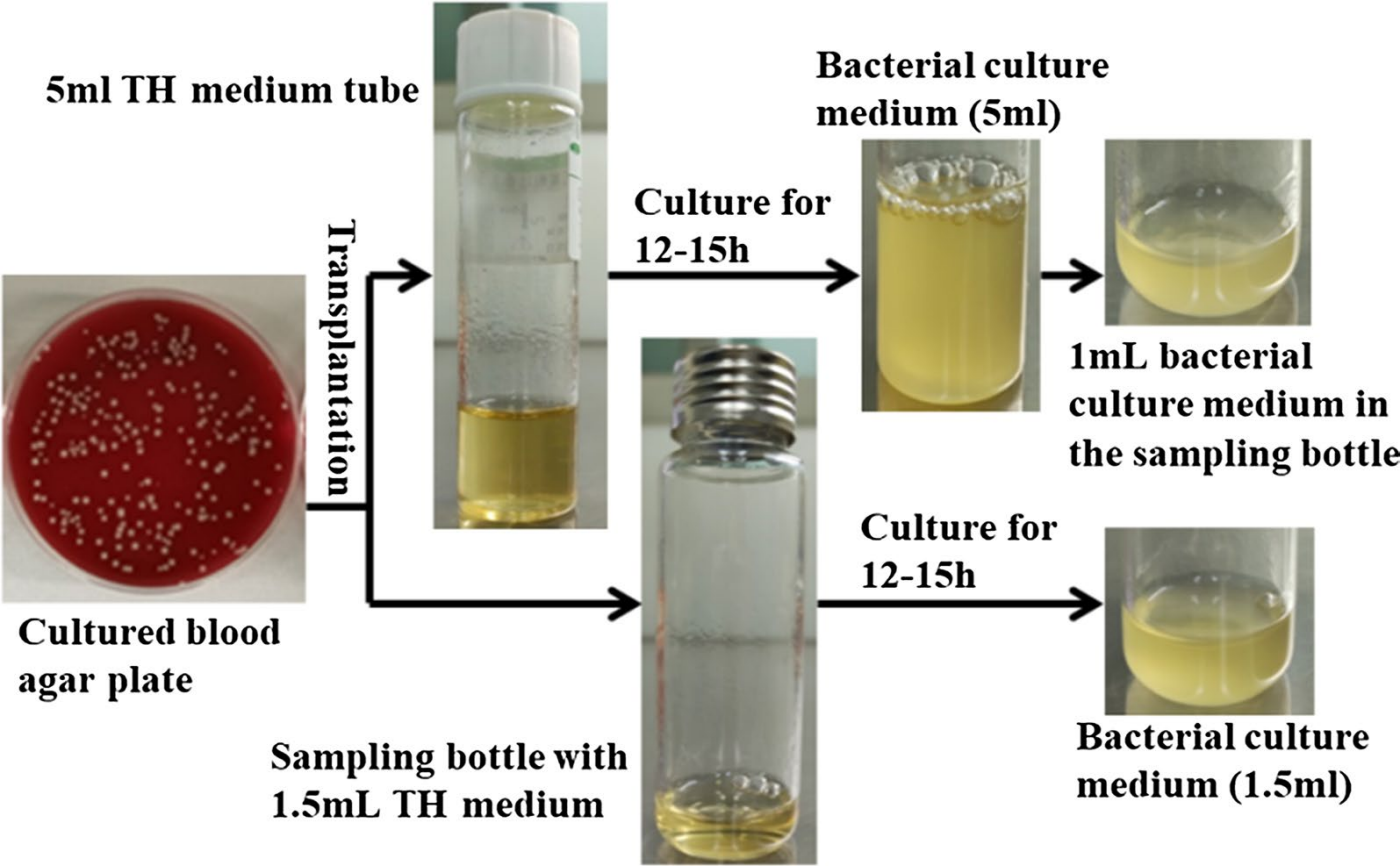
Diagram of the two culture methods

Two sampling methods were used in this study. Initially, the culture was performed in a 5 mL TH medium tube and 1 mL of the culture was transferred to the sampling bottle (20 ml) for detection. The culture was then performed directly in the sampling bottle with 1.5 mL TH medium to facilitate accumulation of mVOCs. Ten tests were conducted for each type of sample. The two culture processes are shown in Fig. 3.

## Results

### Spectrum of samples

**Fig. 4:**
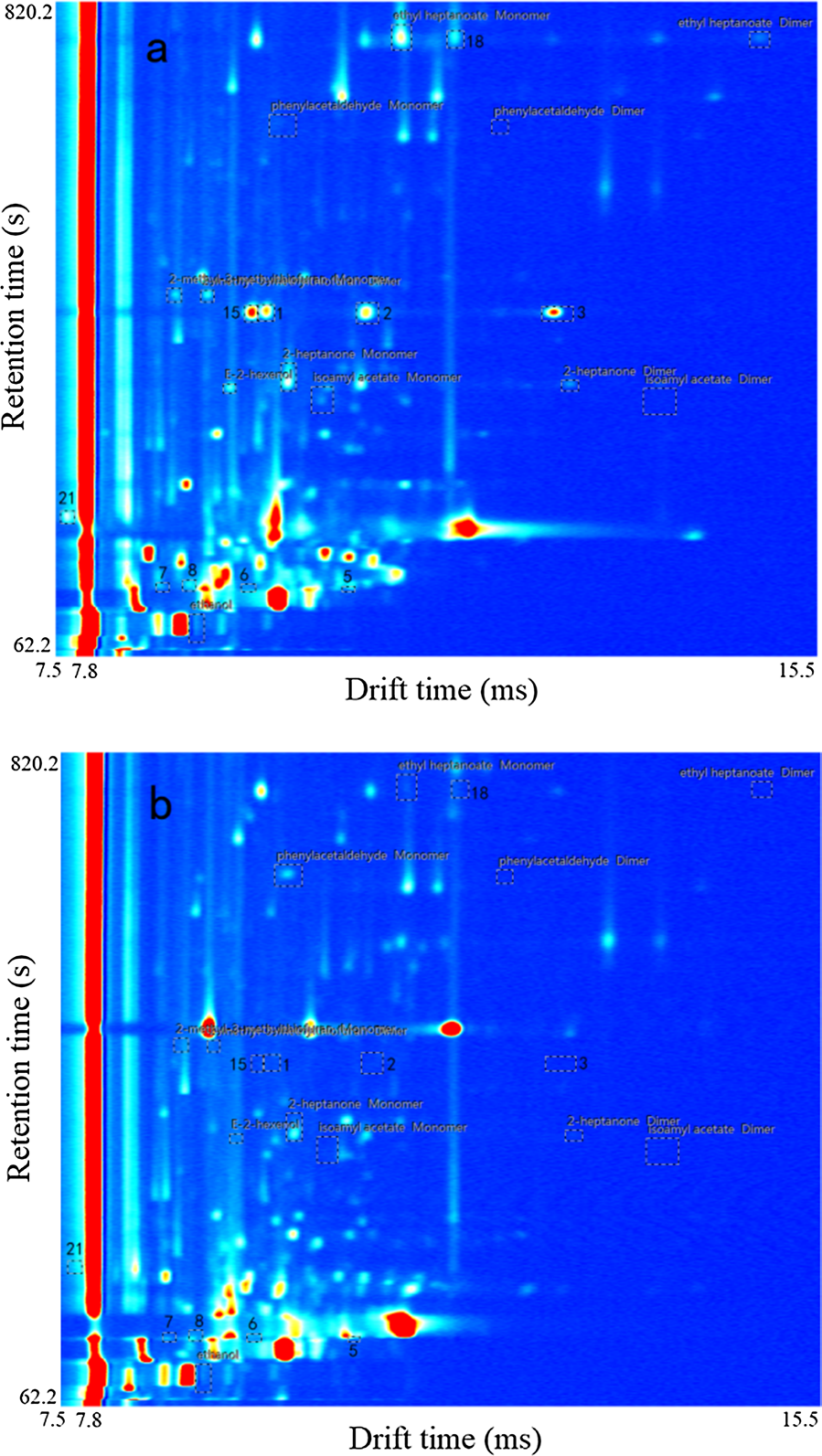
GC-IMS spectra of *P. aeruginosa* and sterile TH medium: **a** Spectrum of *P. aeruginosa* cultured in sampling bottle; **b** Spectrum of sterile TH medium

LAV 2.2.1 (G.A.S, Dortmund, Germany) was used for data analysis. GC-IMS spectra were recorded for all culture samples, with the abscissa for drift time and the ordinate for retention time. Take sterile TH medium and *P. aeruginosa* culture medium as examples (Fig. 4) for GC-IMS spectra. Each bright dot in the blue background represents a volatile organic compound in the sample, and extra-bright areas are represented in yellow and red (see Fig. 4 for example) which mean the higher concentration. The dots are selected in boxes for further statistical analysis. Dots that have been identified were represented by the name of the substances they represent, while the unidentified dots were represented by index numbers. Compared to the background of sterile TH medium, extra characteristic points appeared in all bacterial cultures.

### Specific mVOCs

**Fig. 5:**
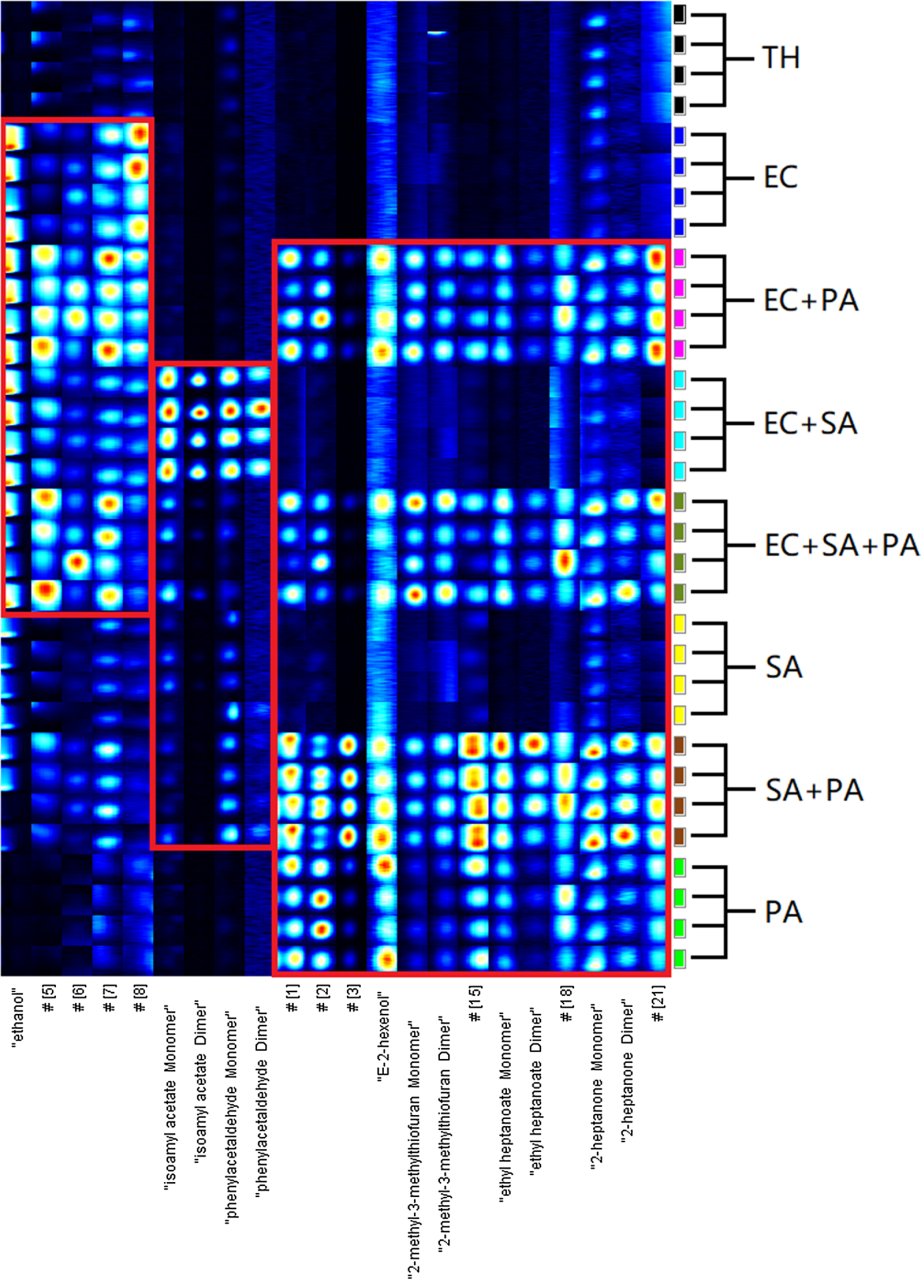
Specific mVOCs of bacterial medium by cultured in sampling bottle. EC, *Escherichia coli*; SA, *Staphylococcus aureus*; PA, *Pseudomonas aeruginosa*; TH, thioglycolate medium

**Fig. 6:**
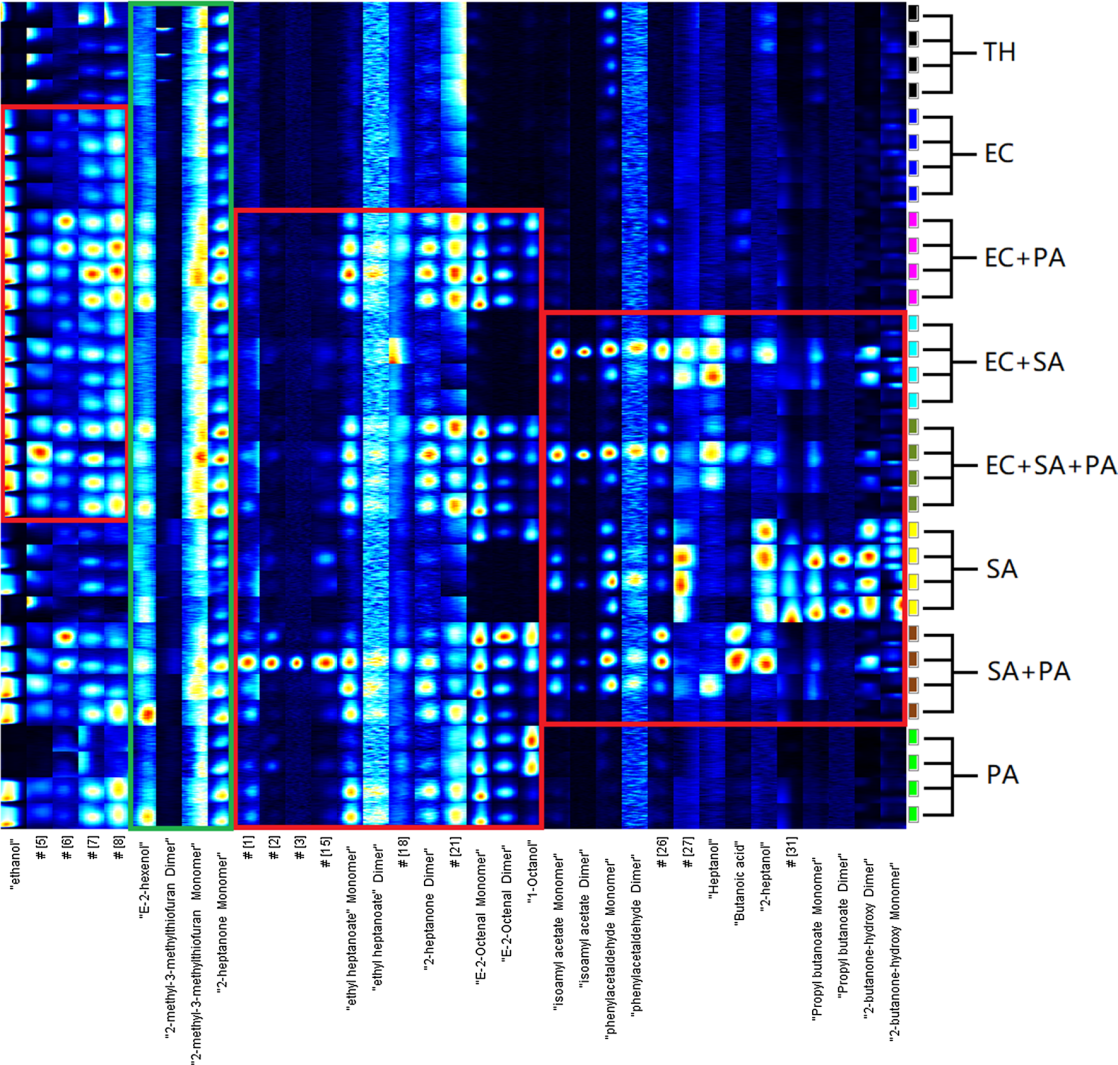
Specific mVOCs of bacterial medium by cultured in TH medium tube. EC, *Escherichia coli*; SA, *Staphylococcus aureus*; PA, *Pseudomonas aeruginosa*; TH, thioglycolate medium

After the significant dots were marked, the following specific mVOCs of bacterial samples can be obtained by comparison with each other and the sterile TH medium (Figs. 5 and 6). To avoid taking too much space, four samples of each type were selected for display. GC × IMS Library Search 1.0.3 (G.A.S, Dortmund, Germany) was used to analyze the characteristic dots qualitatively by comparing with the system database which GC retention time and IMS drift time were both used.

The position of these specific mVOCs can be described by their drift time (Dt) and retention time (Rt), as shown in Table 2. “×” represents that the substance has been detected in this type of sample. For each characteristic dot, peak height (difference between max and min within the box) can be calculated. Based on visual observation, we selected peak height > 0.1 to judge the presence of certain VOC dots. And an mVOC dot is considered present in a type of sample if it is present in any of the 10 repeats. However, some dots with peak height > 0.1 were still excluded since it’s visually clear that the signal was actually from some other substances near the target on the spectrum (Dot #21 in the spectrum of TH, for example). Therefore, the presence of substances should be determined in combination with the peak heights and the GC-IMS spectra.

The drift time of the mVOCs may slightly vary in each test. So the reactive ion peak (RIP, background peak) was used as a reference to normalize the drift time of other mVOCs. Using the representation method in the previous study, the mVOCs that cannot be determined qualitatively is represented by P_x_y in which ‘x’ represents the retention time, and ‘y’ represents the relative drift time.

**Table 2:** Information about specific mVOCs

Dot ID	Compound	Rt(sec)	Dt(RIPrel)	EC	SA	PA	EC + SA	EC + PA	SA + PA	EC + SA + PA	TH
#1	P_462.4_1.24	462.4	1.24			×		×	×	×	
#2	P_462.1_1.38	462.1	1.38			×		×	×	×	
#3	P_461.5_1.63	461.5	1.63			×		×	×	×	
#4	ethanol	94.5	1.15	×	×	×	×	×	×	×	
#5	P_140.1_1.35	140.1	1.35	×	×		×	×	×	×	
#6	P_141.8_1.22	141.8	1.22	×	×	×	×	×	×	×	
#7	P_142.6_1.10	142.6	1.10	×	×		×	×	×	×	×
#8	P_144.5_1.14	144.5	1.14	×	×	×	×	×	×	×	×
#9	isoamyl acetate Dimer	359.1	1.77		×		×			×	
#10	isoamyl acetate Monomer	361.0	1.32		×		×	×	×	×	
#11	Phenylacetaldehyde Monomer	680.8	1.26		×		×		×	×	×
#12	Phenylacetaldehyde Dimer	679.6	1.55				×				
#13	E-2-hexenol	374.4	1.19			×		×	×	×	
#14	2-methyl-3-methylthiofuran Dimer	482.2	1.16			×		×	×	×	
#15	P_461.4_1.22	461.4	1.22		×	×	×	×	×	×	
#16	2-methyl-3-methylthiofuran Monomer	483.0	1.12			×		×	×	×	
#17	ethyl heptanoate Monmer	783.9	1.42			×		×	×	×	
#18	P_781.2_1.49	781.2	1.49			×		×	×	×	
#19	2-heptanone Dimer	377.6	1.65	×	×	×	×	×	×	×	×
#20	ethyl heptanoate Dimer	781.2	1.90			×		×	×	×	
#21	P_224.1_0.98	224.1	0.98			×		×	×	×	
#22	2-heptanone Monomer	387.6	1.28	×	×	×	×	×	×	×	×
#23	E-2-Octenal Monomer	723.9	1.34	×	×	×	×	×	×	×	×
#24	1-Octanol	718.4	1.47			×		×	×	×	
#25	E-2-Octenal Dimer	714.4	1.84			×		×	×	×	
#26	P_358.7_1.43	358.7	1.43		×	×	×	×	×	×	
#27	P_451.5_1.08	451.5	1.08		×		×		×	×	
#28	Heptanol	466.4	1.40				×		×	×	
#29	Butanoic acid	321.1	1.39		×	×	×	×	×	×	
#30	2-heptanol	417.5	1.38		×		×		×	×	
#31	P_234.4_1.34	234.4	1.34		×		×		×	×	
#32	Propyl butanoate Monomer	424.0	1.26		×		×		×	×	×
#33	Propyl butanoate Dimer	417.7	1.68		×		×		×		
#34	2-butanone-hydroxy Dimer	191.5	1.34	×	×	×	×	×	×	×	×
#35	2-butanone-hydroxy Monomer	218.3	1.07	×	×	×	×	×	×	×	×

To evaluate the statistical value of a certain mVOC to identify certain bacteria, for example, EC, we compared the peak values of that mVOC in cultures containing EC and those just short of EC (EC vs. TH, EC + SA vs. SA, EC + PA vs. PA, EC + SA + PA vs. SA + PA) by Kruskal-Walis test (the distribution of sample data does not conform to normal distribution and homogeneity of variance). The mVOC is considered of statistical value if all four comparisons were statistically significant (*P* < 0.05), the dot ID and the largest *P* value are listed in Table 3.

**Table 3:** Kruskal-Walis test to evaluate the discriminative power of the mVOCs

Comparisons	Culture methods	Dot ID and the largest *P* value in a certain comparison
EC-TH EC + SA-SA EC + PA-PA EC + SA + PA-SA + PA	Direct	#4: 0.002; #5: 0.019; #6: 0.034; #7: 0.003
	Indirect	#4: 0.013; #7: 0.041; #8: 0.023
SA-TH SA + EC-EC SA + PA-PA SA + EC + PA-EC + PA	Direct	#10: 0.002; #11: 0.000
	Indirect	#9: 0.005; #10: 0.049; #11: 0.003; #27: 0.028; #32: 0.010; #34: 0.015; #35: 0.007
PA-TH PA + EC-EC PA + SA-SA PA + EC + SA-EC + SA	Direct	#1 - #3: 0.000; #13: 0.000; #15 - #21: 0.000; #22: 0.004
	Indirect	#17: 0.000; #19: 0.001; #20: 0.006; #23: 0.000; #24: 0.001; #25: 0.000

### Principal component analysis (PCA)

**Fig. 7:**
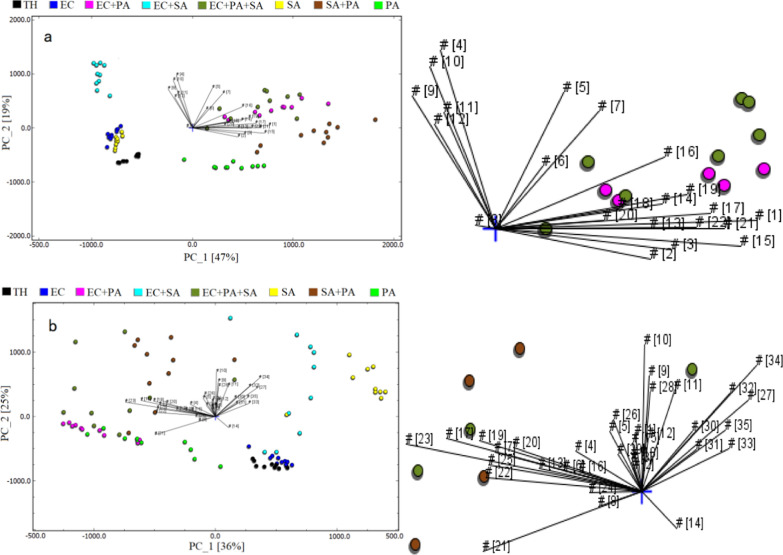
PCA spectra of bacterial medium cultured in: **a** sampling bottle; **b** TH medium tube. EC, *Escherichia coli*; SA, *Staphylococcus aureus*; PA, *Pseudomonas aeruginosa*; TH, thioglycolate medium

Principal component analysis was performed using selected specific mVOCs, as shown in Fig. 7. The font of loading plot cannot be enlarged. Therefore, to facilitate viewing, an enlarged version of this area placed on the right. It can be seen that the separability of the bacterial cultures was higher when the bacteria was cultured directly in the sampling bottle than when it’s cultured initially in TH medium tube then transferred to the sampling bottle. And we can see which substance results in the most variance and which substances are highly correlated.

## Discussion

The purpose of this study was to explore the possibility of rapid identification of compound wound infection bacteria culture according to the mVOCs detected by GC-IMS. Intuitively, for *E.coli*, when cultured directly in the sampling bottle, five mVOCs (#4-#8) increased significantly when *E.coli* was present (Fig. 5). But, if the indirect sampling method was used, the differences were not so obvious. Among them, ethanol (#4) has been reported (Kunze et al. 2013) to reflect its facultative anaerobic metabolism (decomposing glucose by anaerobic fermentation and producing lactic acid, ethanol, etc. as end products) (Clark et al. 1989; Eisenberg et al. 1967). *S. aureus*, an aerobic or facultative anaerobic bacterium (Ezra et al. 1983), can also decompose glucose to produce ethanol, but at a much lower level. Four mVOCs (#9-#12, monomers and dimers of isoamyl acetate and phenylacetaldehyde) seem to be specific for *S. aureus* when the direct sampling method was used. When the indirect sampling method was used, another ten specific mVOCs for *S. aureus* (#26-#35) appeared. However, these 10 mVOCs appeared only in some of the tests and it’s not clear why they didn’t appear in other repeats. As for *P. aeruginosa*, when using the direct sampling method, thirteen mVOCs (#1-#3, #13-#22) clearly differentiate samples containing *P. aeruginosa* from those not. But when the indirect sampling method was used, four mVOCs (#13, #14, #16 and #22) no longer showed significant differences among the types of samples, but another three mVOCs (#23-#25) specific for *P. aeruginosa* appeared.

Results of statistical analysis in Table 3 generally confirmed our intuitive observation in Figs. 5 and 6, but excluded some mVOCs due to high variance in repeated experiments, especially when the indirect method was used. And it should be noted that some mVOCs dimers showed no significant difference in our paired comparisons above, but they shall also have positive diagnostic value if their corresponding monomers do, as these dimmers only appear when the corresponding monomers reach high level.

Acetone (Bunge et al. 2008) and Dimetyl sulphide (Umber et al. 2013) are considered to be specific mVOCs of *E. coli*. However, in our study, acetone was detected in all bacteria samples at similar concentrations, which is consistent with the results of the previous study (Drees et al. 2019). Dimetyl sulphide was not detected by the device in the positive model in our study, but it has been detected using the same device in negative mode (Drees et al. 2019). In addition, indole is generally considered to be the specific mVOC of *E. coli* (Chen et al. 2016; Drees et al. 2019; Kunze et al. 2013), but was not detected in this study. This was likely due to absence of tryptophan in our culture medium, as suggested that *E. coli* produced indole by decomposing tryptophan (Wang et al. 2001).

Several studies identified 3-methyl-butanal as the specific mVOC of *S. aureus* (Drees et al. 2019; Lawal et al. 2018; Thorn et al. 2011). In this study, 3-methyl-butanal was also detected in other samples, even in sterile TH medium, but the concentration was much higher when *S. aureus* was present.

Acetone, azane, dodecane and 2-ethylhexan-1-ol were found related to *P. aeruginosa* (Kunze et al. 2013). As mentioned earlier, under our conditions, acetone cannot be regarded as a specific mVOC. Azane and dodecane could not be detected by our system in principle; while 2-ethylhexan-1-ol appeared in all species without significant differences in concentrations among the samples. The presence of 2-pentanone in the headspace of *E. coli* and *S. aureus* has been reported (Hettinga et al. 2008). In this study, 2-pentanone also appeared in sterile TH medium but only as monomer. When *P. aeruginosa* was present, the concentration of 2-pentanone increased significantly and an obvious dot of its dimer appeared.

Such inconsistency between previous researches can be attributed to differences in culture media, as bacteria may produce different mVOCs when different nutrients were provided. With the medium used in this study, specific mVOCs for identification of the three bacteria were detected. These results suggest the possibility to identify certain bacteria in mixed bacteria by detecting the mVOCs with GC-IMS. The specific mVOCs may depend on the culture medium used in each microbial laboratory.

On the other hand, the sampling bottle is capped with a soft plug, through which, the sampling needle get access to the headspace gas, and 1 mL of the headspace gas is sucked in for detection. Proper amount of sample is added to the sampling bottle so that sufficient amount of bacteria can grow. The liquid level is well below the inserted sampling needle. Initially, the bacteria were cultured in a 5 ml TH medium tube and 1 mL of the culture was transferred to the sampling bottle for detection. It was found during the experiment that mVOCs accumulate in the headspace and some may escape during the transfer, making it more difficult to detect low-concentration mVOCs. So, the bacteria were cultured in the sampling bottle itself for direct sampling. In this way, it is expected that the accumulated mVOCs can be largely retained. It can be seen from the PCA spectra that the direct culture way had higher separability. This indicates that an effective culture method is conducive to the search for bacterial mVOCs. It would be expected that direct culture would have more mVOCs detected than the indirect culture, but the results were on the contrary. It seems that the indirect method was not so stable, as some mVOCs may appear occasionally in 10 repeats. We speculated that moving the culture from TH tube to sample bottle exposes the sample to external environment and brings uncertainty.

In conclusion, GC-IMS technology was used to detect cultures of *E. coli*, *S. aureus* and *P. aeruginosa* alone and in their mixtures. The data were analyzed with the softwares of the system, and the specific mVOCs useful for identification of the three kinds of bacterium were obtained. When using the system for bacterial identification and analysis, better results were achieved by culturing the bacteria directly in the sampling bottle to distinguish the three kinds of bacterium, as suggested by the PCA results. In addition, culturing the bacteria directly in the sampling bottle also facilitates automation with the automatic sampler.

In this study, we have preliminarily investigated the possibility for rapid identification of certain bacterium in mixed culture of up to three bacteria, and the results was encouraging. However, clinical wound infections are often more complex, with diverse sample types, much more bacteria strains, and much more influencing factors (such as medication, nutritional and immuno status). The discriminative power we have achieved is far from enough. Sampling methods and experimental condition shall be investigated to better differentiate the bacteria and to better resemble clinical situation. Furthermore, human samples from clinic should be collected and detected to find the characteristic mVOCs that can distinguish microorganism types. In addition, GC-IMS produce tremendous amount of data that may hide valuable information about bacteria in the sample, but we have only taken a little portion that are obvious, more delicate data analysis may reveal extra information about the sample. Meanwhile, the system was slightly affected by carry over, more accurate data processing methods may eliminate its influence. Last, GC-IMS currently can’t identify several characteristic VOCs and can’t detect some substances in principle (for example, alkanes), which may be solved by GC-MS.

## Data Availability

The datasets generated during and/or analysed during the current study are available from the corresponding author on reasonable request.

## Abbreviations

GC-IMS: Gas chromatograph-ion migration spectroscopy
mVOCs: Microorganism volatile organic compounds
MALDI-TOF MS: Matrix assisted laser desorption/ionization-time of flight mass spectrometry
VOC: Volatile organic compounds
FAIMS: High field asymmetric ion mobility spectrum
MCC-IMS: Multi-capillary column-ion mobility spectrometry
BC: Blood cultures
BSI: Blood stream infection
PCA: Principal component analysis
EC: *Escherichia coli*
SA: *Staphylococcus aureus*
PA: *Pseudomonas aeruginosa*
TH: Thioglycolate
Dt: Drift time
Rt: Retention time
RIP: Reactive ion peak
